# Quantifying the contribution of 31 risk factors to the increasing prevalence of diabetes among US adults, 2005–2018

**DOI:** 10.3389/fpubh.2023.1174632

**Published:** 2023-05-04

**Authors:** Yue Huang, Yaqing Xu, Yongxia Qiao, Hui Wang, Victor W. Zhong

**Affiliations:** School of Public Health, Shanghai Jiao Tong University School of Medicine, Shanghai, China

**Keywords:** trends, prevalence, diabetes, risk factors, contribution

## Abstract

**Introduction:**

No study has comprehensively quantified the individual and collective contributions of various risk factors to the growing burden of diabetes in the United States.

**Methods:**

This study aimed to determine the extent to which an increase in the prevalence of diabetes was related to concurrent changes in the distribution of diabetes-related risk factors among US adults (aged 20 years or above and not pregnant). Seven cycles of series of cross-sectional National Health and Nutrition Examination Survey data between 2005–2006 and 2017–2018 were included. The exposures were survey cycles and seven domains of risk factors, including genetic, demographic, social determinants of health, lifestyle, obesity, biological, and psychosocial domains. Using Poisson regressions, percent reduction in the β coefficient (the logarithm used to calculate the prevalence ratio for prevalence of diabetes in 2017–2018 vs. 2005–2006) was computed to assess the individual and collective contribution of the 31 prespecified risk factors and seven domains to the growing burden of diabetes.

**Results:**

Of the 16,091 participants included, the unadjusted prevalence of diabetes increased from 12.2% in 2005–2006 to 17.1% in 2017–2018 [prevalence ratio: 1.40 (95% CI, 1.14–1.72)]. Individually, genetic domain [17.3% (95% CI, 5.4%−40.8%)], demographic domain [41.5% (95% CI, 24.4%−76.8%)], obesity domain [35.3% (95% CI, 15.8%−70.2%)], biological domain [46.2% (95% CI, 21.6%−79.1%)], and psychosocial domain [21.3% (95% CI, 9.5%−40.1%)] were significantly associated with a different percent reduction in β. After adjusting for all seven domains, the percent reduction in β was 97.3% (95% CI, 62.7%−164.8%).

**Conclusion:**

The concurrently changing risk factors accounted for the increasing diabetes prevalence. However, the contribution of each risk factor domain varied. Findings may inform planning cost-effective and targeted public health programs for diabetes prevention.

## Introduction

Diabetes is a growing health concern as a leading cause of mortality and disability (1). Among US adults, the estimated prevalence of diabetes has increased dramatically in recent decades, reaching 14.7% in 2019 (2). Diabetes posed a colossal economic burden, including $237 billion in direct medical costs and $90 billion in lost productivity in 2017 in the United States (3). Hence, understanding factors contributing to the increasing prevalence of diabetes is critical for devising public health interventions for the prevention of diabetes.

Diabetes is a complex multifactorial disease. The growing prevalence of diabetes likely results from temporal changes in both genetic and more substantially non-genetic factors. The increasing prevalence of diabetes coincides with the changing prevalence of certain risk factors for diabetes among US adults. The prevalence of general and abdominal obesity has continued to increase since 1999 (4–7). Accumulating evidence links psychosocial factors, such as depression, long work hours, and sleep disturbance, with diabetes (8, 9). US adults with psychosocial distress have been a growing population (10, 11). Changes in demographic composition due to birth, death, and migration are in part responsible for the rising prevalence of diabetes (12). Social determinants of health (SDOH) are strong predictors of diabetes, and specific dimensions of SDOH, such as health insurance coverage and food security, levels have changed since 1999 (13, 14). Furthermore, many risk factors of diabetes commonly co-occur within an individual (15). However, no study has comprehensively quantified the individual and collective contribution of various risk factors to the growing burden of diabetes in the United States. The lack of quantitative understanding of major contributing risk factors presents significant challenges for devising cost-effective and targeted public health interventions to reverse the trends in the prevalence of diabetes.

Using data from the National Health and Nutrition Examination Survey (NHANES), the primary objective of this study was to determine the extent to which the increase in the prevalence of diabetes between 2005–2006 and 2017–2018 was related to concurrent changes in the distribution of a wide range of risk factors individually and collectively among US adults.

## Materials and methods

### Data collection

NHANES, as a multistage, nationally representative survey of the US non-institutionalized civilian population, has been conducted in 2-year cycles since 1999–2000 (16). Data were collected during in-home interviews and study visits at mobile examination centers. Seven cycles between 2005–2006 and 2017–2018 were included because important risk factors reflecting mental health, sleep habits, and disorders were not collected until 2005–2006. Participants aged 20 years or above were included except pregnant women. Written informed consent was obtained from each participant. This study was approved by the Shanghai Jiao Tong University School of Medicine Public Health and Nursing Research Ethics Review Committee.

### Definition of diabetes

Consistent with the previous NHANES studies, diabetes was defined as having a self-reported diabetes diagnosis, a fasting plasma glucose level of 126 mg/dl or more, or a hemoglobin A1c level of 6.5% or more (17).

### Domains of risk factors for diabetes

Based on the literature review and data accessibility, a range of risk factors were included and categorized into seven domains: genetic, demographic, SDOH, lifestyle, obesity, biological, and psychosocial domains.

#### Genetic domain

As a proxy for genetic predisposition, family history of diabetes (yes/no) was self-reported through the question “Including living and deceased, were any of your blood relatives, including father, mother, sisters, or brothers, ever told by a health professional that they had diabetes?”

#### Demographic domain

Demographic variables included age in years, sex (male/female), and race/ethnicity. Race and ethnicity were self-reported based on fix-category questions and categorized as non-Hispanic White, non-Hispanic Black, Hispanic, and other.

#### SDOH domain

SDOH included marital status, education, income, employment status, country of birth, health insurance type, healthcare access, food security, and number of people living in the household. Marital status was grouped into married, widowed, divorced, separated, never married, and living with a partner. Education level was categorized as less than high school, high school graduate, some college, and college graduate or above. The ratio of family income to poverty was calculated by dividing self-reported family income by the Department of Health and Human Services' poverty guidelines, specific to the family size, appropriate year, and state. Employment status included working at a job or business, with a job or business but not at work, looking for work, and not working at a job or business. Country of birth was recorded as born in the US or elsewhere. Health insurance type was defined as private (including any private health insurance, Medi-Gap, or single service plan), public only (including Medicare, Medicaid, State Children's Health Insurance Program, military healthcare, Indian Health Service, state-sponsored health plan, or other government insurance), and no insurance. Routine place to go for healthcare (yes/no) was used as a surrogate for healthcare access. Food security status was grouped into four categories: full food security, marginal food security, low food security, and very low food security (18). The total number of people in the household was self-reported and used as a continuous variable.

#### Lifestyle domain

Lifestyle variables included diet quality, physical activity, smoking status and amount, alcohol drinking status and amount, and sleep hours. The Healthy Eating Index 2015 (HEI-2015) was a measure of diet quality according to the 2015–2020 Dietary Guidelines for Americans (19). For physical activity, work-related physical activity was not collected before 2007. This study only included leisure-time physical activity. The minutes spent on the vigorous-intensity physical activity was multiplied by 2 and added to the minutes spent on the moderate-intensity physical activity in a typical week to create weekly minutes of moderate-intensity equivalent physical activity (20). Cigarette smoking status and alcohol consumption status were categorized as never, former, and current (21, 22). Daily cigarettes smoked were calculated using the number of smoking days during the past 30 days and the average number of cigarettes smoked on the smoking days. Daily drinks consumed was calculated using the number of drinking days during the past 12 months and the average number of alcoholic drinks consumed on the drinking days. Sleep hours at night on weekdays or workdays was self-reported and used as a continuous variable.

#### Obesity domain

Obesity variables included body mass index (BMI) and waist circumference. BMI was computed as weight in kilograms divided by height in meters squared.

#### Biological domain

Biological variables included systolic blood pressure, serum cholesterol, use of four antihypertensive medications associated with developing diabetes (23), and statin use. Systolic blood pressure was calculated by taking the mean of all available measurements. Total cholesterol and high-density lipoprotein cholesterol levels were measured using standard protocols based on the Centers for Disease Control and Prevention's Lipid Standardization Program. Currently taking prescribed angiotensin-converting enzyme inhibitors, angiotensin II receptor blockers, β blockers, thiazides, and statins were determined by trained interviewers who documented the product name from the medication containers. Other biological risk factors, including diastolic blood pressure, uric acid, and estimated glomerular filtration rate, were further contained in the alternative biological domain and evaluated separately in a sensitivity analysis because there is evidence that these factors could be bidirectionally associated with diabetes.

#### Psychosocial domain

Psychosocial variables included working hours, trouble sleeping, and depression symptoms. Hours worked last week was self-reported. Having trouble sleeping (yes/no) was assessed by the response to “Have you ever told a doctor or other health professional that you have trouble sleeping?” The Patient Health Questionnaire-9 was administered to assess the severity of depressive symptoms over the past 2 weeks. It had nine items with four response levels (not at all, several days, more than half the days, nearly every day) scoring from 0 to 3 for each, resulting in a total score of 0 (low depressive symptomatology) to 27 (high depressive symptomatology).

### Statistical analysis

Proportions or means were estimated to describe the characteristics of participants, as appropriate for all risk factors. Logistic regressions for categorical risk factors and linear regressions for continuous risk factors were used to compute crude *P*-value for trend from 2005–2006 to 2017–2018 (24).

The previous study revealed a linear trend in prevalence of diabetes between 1999–2000 and 2017–2018 (17). The linear trend between 2005–2006 and 2017–2018 was confirmed in this study. Poisson regressions were used to estimate the prevalence ratio (PR) for prevalence of diabetes comparing 2017–2018 with 2005–2006 (25). The extent to which the increase in prevalence of diabetes between 2005–2006 and 2017–2018 was related to the pre-specified risk factors or risk factor domains was estimated by calculating percent reduction in the β coefficient for the survey cycle (2017–2018 vs. 2005–2006) on the log-scale. Percent reduction in the β coefficient was obtained by contrasting the two models under comparison: (β_ref_−β_adj_)/β_ref_×100%. β_ref_ was from the base model. β_adj_ was from the model including one or more risk factors or risk factor domains compared with the base model. The 95% confidence intervals (95% CIs) were estimated by performing bootstrap resampling (*n* = 200) (26).

Modeling strategies were described as follows. First, to assess the contribution of individual risk factor domains, the model with adding each of the 31 risk factors was compared with the base model without including any risk factors. Second, to assess the contribution of individual risk factor domains, the model with adding each of the seven risk factor domains was compared with the aforementioned base model. Third, to assess the collective contribution of two or more risk factor domains, each of the seven risk factor domains was sequentially added to the previous model, until all seven domains were included simultaneously. According to the modifiability and etiological proximity of risk factors in regard to diabetes, genetic, demographic, SDOH, lifestyle, obesity, biological, and psychosocial domains were added sequentially. Fourth, to assess the remaining contribution of each risk factor domain, the model excluding one of the seven risk factor domains was compared with the base model. Fifth, to assess the respective contribution of non-modifiable and modifiable risk factors, the model adjusting for non-modifiable risk factor domains (genetic and demographic domains) and modifiable risk factor domains (all other five domains) was compared with the base model. To conservatively account for possible non-linear associations between risk factors and diabetes, a quadratic term was added for all risk factors in continuous form.

Missing data were imputed with multiple imputation by chained equations (27). Considering the convergence issues of logistic regression models, multi-categorical risk factors were converted to binary ones and treated as continuous variables. Instead of the linear regression approach, predictive mean matching was chosen for the estimation, given its advantage of better preserving the original distribution of data (28). The number of nearest donors in the matching pool was set to be 10 (29). According to the recommendations that the number of imputations should at least equal the highest percentage of the fraction of missing information (FMI), the number of imputed datasets was set to be 10 because the highest FMI percentage of an individual variable was < 10% (27). Each model was executed within each of the 10 imputed datasets to obtain 10 sets of estimates, which were then meta-analyzed to produce one pooled estimate. A sensitivity analysis was conducted by performing a complete case analysis to assess the robustness of primary results.

Based on the least-common denominator rule, reconstructed weights incorporating weights from the dietary 2-day sample and fasting subsample were used to ensure the representativeness of the estimates. Design variables were further adjusted to obtain unbiased estimates and standard errors. All analyses were implemented with SAS version 9.4 and STATA version 17.0. A two-tailed *P-value* of < 0.05 denoted statistical significance.

## Results

**Figure 1:**
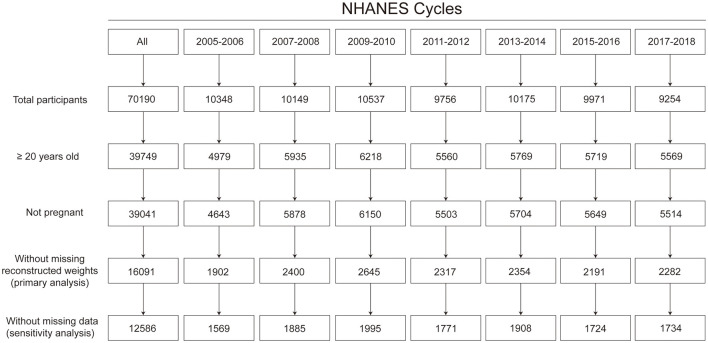
Flow chart for sample size. Based on the least-common denominator rule, reconstructed weights incorporating weights from the dietary 2-day sample and fasting subsample were used to ensure the representativeness of the estimates.

Among the 16,091 participants included, 3,505 (21.8%) participants had missing information on the outcome or risk factors of interest (Figure 1). After multiple imputation, the weighted mean age was 48.3 years, 47.8% were men, and 68.5% were non-Hispanic White.

The estimated crude prevalence of diabetes increased significantly from 12.2% (95% CI, 10.1%−14.3%) in 2005–2006 to 17.1% (95% CI, 15.2%−18.9%) in 2017–2018 [crude prevalence ratio (PR): 1.40 (95% CI, 1.14–1.72)].

### Crude trends in risk factors

The estimated proportions of participants having a family history of diabetes, having multi-racial backgrounds, looking for work, with marginal, low or very low food security, having public insurance only, never smoking, drinking currently, taking β blockers, taking statins, and having trouble sleeping increased significantly between 2005–2006 and 2017–2018 (all *P* for trend < 0.05). The estimated proportions of participants having non-Hispanic White background, with an education level of less than high school, with full food security, having private insurance, having no insurance, having routine place to go for healthcare, and smoking currently decreased significantly between 2005–2006 and 2017–2018 (all *P* for trend < 0.05). The estimated means of participants' age, sleep hours, BMI, waist circumference, systolic blood pressure, and depression score increased significantly from 2005–2006 to 2017–2018 (all *P* for trend < 0.05). The estimated means of participants' leisure-time physical activity level, daily cigarettes smoked, total cholesterol level, and hours worked during the last week decreased significantly from 2005–2006 to 2017–2018 (all *P* for trend < 0.05; Table 1).

**Table 1:** Participant characteristics, 2005–2018^a^.

**Characteristics**	**2005– 2006**	**2007– 2008**	**2009– 2010**	**2011– 2012**	**2013– 2014**	**2015– 2016**	**2017– 2018**	***P* for trend**
No. of participants^b^	1,902	2,400	2,645	2,317	2,354	2,191	2,282	
**Genetic domain**								
Family history of diabetes^c^, %	41.6	38.6	37.5	34.9	38.5	45.1	47.7	0.001
**Demographic domain**								
Age, years	47.5	47.7	47.7	48.2	48.3	49.2	49.1	0.03
Male, %	47.8	48.2	47.9	47.6	47.5	47.1	48.5	0.94
Race and ethnicity^d^, %								
Non-Hispanic White	72.7	72.3	69.1	68.2	66.8	66.6	64.7	0.02
Non-Hispanic Black	10.9	10.7	10.8	11.0	11.4	11.2	11.4	0.75
Hispanic	11.3	12.2	13.4	13.8	14.5	13.5	14.2	0.24
Other	5.1	4.8	6.6	6.9	7.4	8.7	9.6	< 0.001
**Social determinants of health domain**								
Marital status, %								
Married	57.9	58.0	57.2	55.9	58.7	56.5	51.8	0.07
Widowed	5.9	6.5	6.3	5.2	5.9	5.9	5.9	0.65
Divorced	10.1	9.4	9.9	10.5	10.2	9.8	12.8	0.08
Separated	2.5	2.6	1.9	2.2	2.0	2.1	2.4	0.69
Never married	15.0	16.6	17.3	18.0	17.0	16.7	17.4	0.38
Living with partner	8.6	6.9	7.4	8.2	6.1	9.1	9.7	0.32
Education level, %								
Less than high school	15.6	18.2	17.9	16.7	15.1	13.4	10.2	< 0.001
High school graduate	26.0	24.9	21.8	19.9	20.4	22.9	27.1	0.93
Some college	32.7	28.3	29.6	31.5	33.0	30.8	32.1	0.52
College graduate or above	25.7	28.7	30.7	31.8	31.6	32.9	30.6	0.13
Employment status, %								
Working at a job or business	63.4	61.4	59.1	59.7	58.4	58.7	59.4	0.07
With a job or business but not at work	3.5	2.7	2.6	1.7	1.5	3.2	2.1	0.15
Looking for work	1.2	1.9	3.9	4.4	2.7	3.3	3.2	0.003
Not working at a job or business	31.9	34.0	34.5	34.2	37.4	34.7	35.2	0.15
Ratio of family income to poverty	3.1	3.1	2.9	2.9	2.9	3.0	3.0	0.31
Born in 50 US states or Washington, DC, %	87.1	86.0	81.3	83.2	83.7	83.5	82.9	0.12
Total number of people in the household	2.9	2.9	3.1	3.0	3.1	3.0	3.0	0.26
Food security^e^, %								
Full food security	84.3	82.7	78.5	75.0	76.4	71.9	69.3	< 0.001
Marginal food security	7.6	7.0	8.4	9.2	9.4	11.2	11.8	< 0.001
Low food security	5.4	6.8	7.6	9.0	8.3	9.7	9.8	< 0.001
Very low food security	2.8	3.5	5.5	6.8	6.0	7.2	9.2	< 0.001
Health insurance type, %								
Private	67.4	69.5	64.9	61.3	61.7	65.3	60.2	0.01
Public only	14.6	14.3	14.8	19.4	19.9	22.3	26.6	< 0.001
Uninsured	17.9	16.2	20.2	19.3	18.3	12.3	13.2	0.02
Routine place to go for healthcare, %	86.0	86.7	87.0	86.1	84.2	84.4	81.7	0.004
**Lifestyle domain**								
Healthy Eating Index 2015 score	52.4	53.4	54.3	55.2	54.3	53.1	51.9	0.46
Leisure-time physical activity^f^, min/week	298.3	209.4	197.7	218.1	197.5	202.1	216.1	0.002
Cigarette smoking status, %								
Never	49.1	53.0	55.9	56.3	56.0	54.1	56.6	0.02
Former	25.8	24.5	25.3	23.9	25.2	26.4	25.8	0.63
Current	25.1	22.5	18.8	19.8	18.8	19.5	17.6	0.001
Daily cigarettes smoked	3.9	3.5	2.3	2.4	2.1	2.2	2.0	< 0.001
Alcohol consumption status, %								
Never	10.7	11.5	10.8	9.9	12.7	12.4	6.4	0.14
Former	17.6	18.0	15.6	15.4	14.5	14.2	16.4	0.08
Current	71.7	70.5	73.7	74.8	72.9	73.4	77.2	0.02
Daily drinks consumed	0.6	0.4	0.5	0.5	0.4	0.5	0.5	0.25
Sleep hours at night	6.8	6.9	6.9	6.9	6.9	7.7	7.6	< 0.001
**Obesity domain**								
Body mass index^g^, kg/m^2^	28.9	28.6	29.0	29.0	29.6	29.8	29.7	< 0.001
Waist circumference, cm	98.5	98.5	99.2	99.3	100.6	101.7	101.2	< 0.001
**Biological domain**								
Systolic blood pressure, mm Hg	122.7	120.6	119.4	121.3	121.3	123.2	123.3	0.005
Taking angiotensin-converting enzyme inhibitors, %	10.9	12.4	13.3	13.3	14.1	14.6	11.2	0.26
Taking angiotensin II receptor blockers, %	5.8	8.0	6.7	5.4	6.9	8.0	7.5	0.18
Taking β blockers, %	11.2	11.2	11.9	12.2	11.5	12.2	15.4	0.01
Taking thiazides, %	9.2	8.8	9.4	10.3	9.5	8.9	7.8	0.42
Total cholesterol, mg/dl	198.2	196.2	195.3	194.5	189.5	191.5	187.3	< 0.001
High-density lipoprotein cholesterol, mg/dl	55.6	52.9	53.9	53.3	53.6	55.7	53.9	0.86
Taking statins, %	14.8	17.5	18.4	19.4	21.4	20.8	20.7	< 0.001
**Psychosocial domain**								
Hours worked last week	26.8	26.0	23.8	23.8	23.7	23.5	23.9	0.002
Depression score^h^	2.5	2.9	3.1	3.0	3.0	3.2	3.2	< 0.001
Have trouble sleeping, %	25.0	25.7	25.9	27.6	28.8	30.9	34.3	< 0.001

^a^Data are presented as proportions for categorical variables and means for continuous variables.

^b^Unweighted sample size.

^c^Including living and deceased, any blood relatives, including grandparents, parents, sisters, or brothers, were ever told by a health professional that they had diabetes.

^d^Race and ethnicity were determined by self-report in fixed categories.

^e^Adult food security status was measured through the US Household Food Security Survey Module, of which 10 questions for the adults in the household were used to create four response levels, based on the number of affirmative responses to these questions.

^f^The minutes spent on the vigorous-intensity physical activity was multiplied by two and added to the minutes spent on the moderate-intensity physical activity in a typical week to create weekly minutes of moderate-intensity equivalent physical activity.

^g^Body mass index was computed as weight in kilograms divided by height in meters squared.

^h^Patient Health Questionnaire-9 was used to assess the severity of depressive symptoms over the past 2 weeks. The questionnaire has nine items with each having four response levels (not at all, several days, more than half the days, nearly every day) and scoring from 0 to 3. The total score ranges from 0 (low depressive symptomatology) to 27 (high depressive symptomatology).

### Contribution of risk factors to the growing prevalence of diabetes

Individually, adjusting for family history of diabetes [17.3% (95% CI, 5.4%−40.8%)], age [25.1% (95% CI, 8.4%−49.5%)], race and ethnicity [5.9% (95% CI, 1.8%−12.6%)], education level [−12.7% (95% CI, −27.3% to −5.8%)], food security [8.7% (95% CI, 3.3%−21.0%)], health insurance type [25.7% (95% CI, 15.6%−47.9%)], routine place to go for healthcare [−8.5% (95% CI, −20.7% to −1.5%)], HEI-2015 [−2.0% (95% CI, −6.3% to −0.1%)], leisure-time physical activity [16.4% (95% CI, 7.4%−34.0%)], BMI [20.4% (95% CI, 5.6%−44.0%)], waist circumference [33.6% (95% CI, 14.7%−66.8%)], taking β blockers [18.5% (95% CI, 4.7%−37.1%)], total cholesterol [15.9% (95% CI, 8.3%−31.7%)], high-density lipoprotein cholesterol [12.9% (95% CI, 1.6%−28.1%)], statins use [34.6% (95% CI, 17.6%−61.7%)], depression score [9.1% (95% CI, 4.7%−18.9%)], or having trouble sleeping [12.4% (95% CI, 6.8%−22.2%)] was associated with a significant percent reduction in the β coefficient when comparing prevalence of diabetes between 2017–2018 and 2005–2006 (Table 2).

**Table 2:** Prevalence ratios for contrasting diabetes prevalence in 2017–2018 vs. 2005–2006 and percent reduction in β estimates according to individual risk factors.

**Risk factors**	**Prevalence ratio (95% CI)**	**Percent reduction in β (95% CI), %^a^**
Base model	1.40 (1.14–1.72)	[Reference]
**Individual adjustment for each risk factor**		
Family history of diabetes	1.32 (1.10–1.59)	17.3 (5.4 to 40.8)
Age	1.29 (1.07–1.55)	25.1 (8.4 to 49.5)
Sex	1.40 (1.14–1.72)	0.3 (−2.0 to 2.2)
Race and ethnicity	1.37 (1.12–1.69)	5.9 (1.8 to 12.6)
Marital status	1.42 (1.16–1.74)	−3.8 (−16.4 to 5.8)
Education level	1.46 (1.21–1.77)	−12.7 (−27.3 to −5.8)
Employment status	1.35 (1.14–1.61)	9.9 (−1.5 to 22.1)
Ratio of family income to poverty	1.39 (1.13–1.70)	3.0 (−1.6 to 9.2)
Country of birth	1.40 (1.14–1.71)	1.2 (0.1 to 3.5)
Total number of people in the household	1.41 (1.15–1.73)	−2.4 (−9.8 to 4.4)
Food security	1.36 (1.11–1.67)	8.7 (3.3 to 21.0)
Health insurance type	1.28 (1.05–1.57)	25.7 (15.6 to 47.9)
Routine place to go for healthcare	1.44 (1.18–1.76)	−8.5 (−20.7 to −1.5)
Healthy Eating Index 2015 score	1.41 (1.15–1.74)	−2.0 (−6.3 to −0.1)
Leisure-time physical activity	1.33 (1.09–1.62)	16.4 (7.4 to 34.0)
Cigarette smoking	1.42 (1.16–1.74)	−3.7 (−14.0 to 4.1)
Alcohol consumption	1.43 (1.17–1.75)	−6.8 (−21.6 to 3.7)
Sleep hours at night	1.36 (1.11–1.67)	8.7 (−0.1 to 21.0)
Body mass index	1.31 (1.09–1.58)	20.4 (5.6 to 44.0)
Waist circumference	1.25 (1.04–1.50)	33.6 (14.7 to 66.8)
Systolic blood pressure	1.37 (1.15–1.65)	5.6 (−15.8 to 17.2)
Taking angiotensin-converting enzyme inhibitors	1.39 (1.15–1.69)	1.7 (−14.3 to 15.5)
Taking angiotensin II receptor blockers	1.36 (1.13–1.64)	8.8 (−0.6 to 20.5)
Taking β blockers	1.32 (1.09–1.59)	18.5 (4.7 to 37.1)
Taking thiazides	1.42 (1.18–1.72)	−4.9 (−17.5 to 3.5)
Total cholesterol	1.33 (1.08–1.63)	15.9 (8.3 to 31.7)
High-density lipoprotein cholesterol	1.34 (1.09–1.65)	12.9 (1.6 to 28.1)
Taking statins	1.25 (1.03–1.51)	34.6 (17.6 to 61.7)
Hours worked last week	1.36 (1.13–1.63)	9.7 (−0.1 to 21.9)
Depression score	1.36 (1.11–1.67)	9.1 (4.7 to 18.9)
Have trouble sleeping	1.34 (1.09–1.65)	12.4 (6.8 to 22.2)

^a^Percent reduction in the β coefficient, an estimate to quantify the percent contribution of individual risk factors to the increasing prevalence of diabetes comparing 2017–2018 with 2005–2006, was obtained through contrasting the two models under comparison: (β_ref−_β_adj_)/$\beta_{\text{ref}}^{*}$100%. β_ref_ was based on the base model which is a crude Poisson model not adjusted for any domains of risk factors. β_adj_ was based on the model including individual risk factors compared with the base model. The 95% confidence intervals (95% CIs) were estimated by performing bootstrap resampling (n = 200). To conservatively account for possible non-linear associations between risk factors and diabetes, a quadratic term was added for all the risk factors in continuous form.

### Contribution of risk factor domains to the growing prevalence of diabetes

Individually, adjusting for biological domain [46.2% (95% CI, 21.6%−79.1%)], demographic domain [41.5% (95% CI, 24.4%−76.8%)], obesity domain [35.3% (95% CI, 15.8%−70.2%)], psychosocial domain [21.3% (95% CI, 9.5%−40.1%)], or genetic domain [17.3% (95% CI, 5.4%−40.8%)] was associated with significant percent reduction in the β coefficient when comparing prevalence of diabetes between 2017–2018 and 2005–2006 (Table 3).

**Table 3:** Prevalence ratios for contrasting diabetes prevalence in 2017–2018 vs. 2005–2006 and percent reduction in β estimates according to individual domains.

**Models**	**Prevalence ratio (95% CI)**	**Percent reduction in β (95% CI), %^a^**
Base model^b^	1.40 (1.14–1.72)	[Reference]
**Individual adjustment for each domain**		
Base + genetic domain	1.32 (1.10–1.59)	17.3 (5.4 to 40.8)
Base + demographic domain	1.22 (1.02–1.45)	41.5 (24.4 to 76.8)
Base + social determinants of health domain	1.39 (1.17–1.66)	2.0 (−19.1 to 19.4)
Base + lifestyle domain	1.36 (1.12–1.66)	8.0 (−11.4 to 30.3)
Base + obesity domain	1.24 (1.04–1.49)	35.3 (15.8 to 70.2)
Base + biological domain	1.20 (1.02–1.41)	46.2 (21.6 to 79.1)
Base + psychosocial domain	1.30 (1.08–1.57)	21.3 (9.5 to 40.1)
**Sequential adjustment for each domain**		
Base + genetic domain	1.32 (1.10–1.59)	17.3 (5.4 to 40.8)
Further including demographic domain	1.15 (0.97–1.36)	59.0 (36.2 to 112.4)
Further including social determinants of health domain	1.15 (0.97–1.36)	59.1 (33.4 to 107.7)
Further including lifestyle domain	1.12 (0.95–1.32)	67.2 (38.5 to 129.1)
Further including obesity domain	1.08 (0.93–1.25)	78.3 (49.1 to 150.3)
Further including biological domain	1.01 (0.88–1.17)	95.7 (62.7 to 163.2)
Further including psychosocial domain	1.01 (0.87–1.17)	97.3 (62.7 to 164.8)
**Adjustment for all domains but excluding one domain**		
Excluding genetic domain	1.03 (0.89–1.20)	90.1 (57.9 to 154.9)
Excluding demographic domain	1.09 (0.95–1.27)	73.4 (42.5 to 124.5)
Excluding social determinants of health domain	1.01 (0.87–1.17)	97.2 (65.6 to 168.4)
Excluding lifestyle domain	1.01 (0.88–1.17)	96.2 (64.0 to 154.9)
Excluding obesity domain	1.03 (0.87–1.21)	92.2 (57.9 to 159.7)
Excluding biological domain	1.06 (0.91–1.24)	81.7 (52.8 to 153.1)
Excluding psychosocial domain	1.01 (0.88–1.17)	95.7 (62.7 to 163.2)
**Adjustment for non-modifiable and modifiable domains**		
Base + non-modifiable domains	1.15 (0.97–1.36)	59.0 (36.2 to 112.4)
Base + modifiable domains	1.12 (0.96–1.31)	64.7 (40.1 to 110.0)

^a^Percent reduction in the β coefficient, an estimate to quantify the percent contribution of individual and collective domains of risk factors to the increasing prevalence of diabetes comparing 2017–2018 to 2005–2006, was obtained through contrasting the two models under comparison: (β_ref__−_β_adj_)/$\beta_{\text{ref}}^{*}$100%. β_ref_ was based on the base model. β_adj_ was based on the model including one or more risk factor domains compared with the base model. The 95% confidence intervals (95% CIs) were estimated by performing bootstrap resampling (n = 200). To conservatively account for possible non-linear associations between risk factors and diabetes, a quadratic term was added for all the risk factors in continuous form.

^b^Base model is the crude Poisson model not adjusted for any domains of risk factors.

Sequentially, after adjusting for genetic and demographic domains, the percent reduction in the β coefficient was 59.0% (95% CI, 36.2%−112.4%), and the PR for comparing prevalence of diabetes in 2017–2018 with 2005–2006 was no longer significant [PR: 1.15 (95% CI, 0.97–1.36)]. After adjusting for all seven domains of risk factors, the percent reduction in the β coefficient was 97.3% (95% CI, 62.7%−164.8%; Table 3).

When adjusting for all domains but omitting one, the exclusion of demographic domain was associated with the least attenuation in the β coefficient [73.4% (95% CI, 42.5%−124.5%); Table 3]. Percent reduction in the β coefficient was 64.7% (95% CI, 40.1%−110.0%) when adjusting for modifiable domains and 59.0% (95% CI, 36.2%−112.4%) for non-modifiable domains.

### Sensitivity analysis

The percent reduction in the β coefficient when adjusting for the alternative biological domain was 36.8% (95% CI, 11.6–68.7%; Supplementary Table S1). Of the 12,586 participants with complete information, the unadjusted prevalence of diabetes increased significantly from 2005–2006 (11.1%) to 2017–2018 [16.1%; PR: 1.46 (95% CI, 1.12–1.90)]. The contribution of a single risk factor was quantified (Supplementary Table S2). Individually, genetic, demographic, obesity, biological, or psychosocial domain was associated with a significant reduction in the β coefficient (Supplementary Table S3). These results were materially similar to the results of primary analysis using imputed data sets.

## Discussion

Among US adults, the estimated prevalence of diabetes increased significantly in parallel with concurrent changes in the distribution of a comprehensive set of non-modifiable and modifiable risk factors for diabetes from 2005–2006 to 2017–2018. Ranked by the magnitude of contribution, the increasing prevalence of diabetes was significantly related to biological, demographic, obesity, psychosocial, and genetic domains (ranging from 46% to 17%). After taking into account all seven risk factor domains, the increasing trend in prevalence of diabetes was no longer observed. These findings provide concrete, informative, and targeted data for guiding future public health efforts for the prevention of diabetes.

The demographic domain had a major contribution to the increasing prevalence of diabetes, which primarily resulted from aging and increasing proportion of racial and ethnic minorities. These trends in the demographic composition of the US population likely continue and further contribute to the growing burden of diabetes (12). As this study found that ~40% of the increase in the diabetes prevalence was related to changing demographic factors, further interventions targeting the aging population and ethnic minorities should be emphasized to effectively address the growing burden of diabetes among US adults.

Family history of diabetes is a well-established strong risk factor for diabetes (30, 31). As a proxy for genetic predisposition, its prevalence was speculated to be relatively stable in short periods. However, an increasing trend in the family history of diabetes was observed in this study, which may in part be driven by aging. Furthermore, the increase in genetically susceptible individuals in the gene pool could be caused by the increase in racial and ethnic minorities (32). The demographic and genetic factors are considered non-modifiable but contributed to a substantial proportion of the growing diabetes burden.

The biological domain accounted for the greatest proportion of the increasing prevalence of diabetes. Biological factors are most proximal to diabetes onset, and risk factors from other domains may directly and indirectly influence biological factors. The prevalence of statin use, the strongest contributor to the rising prevalence of diabetes within the biological domain, increased significantly. Statins are associated with accelerated progression to diabetes via the mechanisms of insulin secretion, insulin resistance, and cellular metabolisms of glucose (33, 34). In addition, the prevalence of taking β blockers, a cardioprotective drug that could worsen glycemic control by increasing insulin resistance and decreasing insulin release (35), also increased significantly, which contributed to the increasing prevalence of diabetes.

Approximately one-third of the increasing prevalence of diabetes was related to elevating BMI and waist circumference; the latter made a greater contribution. BMI and waist circumference increased parallel with a prevalence of diabetes (7, 17). Studies have implied that waist circumference was a stronger predictor for diabetes, especially among persons of low or normal weight compared with BMI (36, 37). Obesity appears to be a mediating factor connecting upstream genetic and lifestyle risk factors and downstream biological risk factors. Therefore, obesity can be a pivotal intervention target from the public health perspective for diabetes prevention (38).

Psychological distress, depression, and sleep disturbance are risk factors for diabetes, especially among the subpopulation with prediabetes and other risk factors (39, 40), but their contribution to diabetes burden has not been well-quantified. The mean depression score and prevalence of trouble sleeping increased significantly among the study population, and both had a significant contribution to the rising prevalence of diabetes. Previous evidence has indicated an increasing prevalence of psychological distress among US adults, especially among young adults (10, 11), of whom the diabetes burden also increased dramatically (17, 41).

SDOH and lifestyle factors are known risk factors for diabetes. The insignificant results for SDOH and lifestyle domains did not translate into that these factors were not important. First, many of these factors, such as diet quality and income, did not change significantly during the study period. Second, the opposite trends in specific risk factors were observed within each domain that contributed negatively to diabetes burden. For example, for the SDOH domain, the contribution by decreased food security level and proportion of the uninsured (i.e., leading to better screening and detection of diabetes) may have been largely counterbalanced by improved education and decreased proportion of people having routine place to go for healthcare.

Non-modifiable factors played an important role in the growing prevalence of diabetes, but modifiable factors from the five domains together accounted for 65% of the increased diabetes prevalence from 2005–2006 to 2017–2018. Through the control of modifiable risk factors, the increasing trend of diabetes can be slowed or even reversed. This analysis precisely identified domains and risk factors of priority for diabetes prevention, which may shed light on the design of effective targeted public health interventions.

### Limitations

This study has several limitations. First, causal inference cannot be made with cross-sectional observational data. Findings of this study provide only suggestive evidence on possible contributors for the increasing burden of diabetes. Second, relying on self-reported data may have led to misclassification of diabetes and risk factors. Third, genetic susceptibility was represented by a convenient proxy family history of diabetes, instead of genetic data. Fourth, some risk factors, such as sedentary activity, low birthweight, C-reactive protein, and urinary cadmium, were not considered because they were not available or collected in subsamples or specific cycles only, or had bidirectional or controversial associations with diabetes. Fifth, the grouping method for risk factor domains was somewhat arbitrary. Sixth, this study focused on quantifying the overall contributions of risk factors to the increasing burden of diabetes. Therefore, subgroup analyses by demographic factors were not conducted because changes in these stratification factors themselves were important contributors.

## Conclusion

Based on the NHANES data, the increasing trend in prevalence of diabetes among US adults between 2005–2006 and 2017–2018 was related to concurrent changes in the distribution of diabetes-related risk factors. Ranked by the magnitude of contribution, biological, demographic, obesity, psychosocial, and genetic domains of risk factors significantly but differentially accounted for the growing prevalence of diabetes.

## Data availability statement

Publicly available datasets were analyzed in this study. This data can be found at: https://wwwn.cdc.gov/nchs/nhanes/Default.aspx.

## Ethics statement

The studies involving human participants were reviewed and approved by Shanghai Jiao Tong University School of Medicine Public Health and Nursing Research Ethics Review Committee. The patients/participants provided their written informed consent to participate in this study.

## Author contributions

YH: conceptualization, formal analysis, methodology, writing—original draft, and writing—review and editing. YX: methodology and writing—original draft. YQ: writing—original draft. HW: conceptualization, resources, supervision, and writing—review and editing. VZ: conceptualization, formal analysis, methodology, writing—original draft, resources, supervision, and writing—review and editing. All authors contributed to the article and approved the submitted version.
