# Supplementary material for: Quantifying the contribution of 31 risk factors to the increasing prevalence of diabetes among US adults, 2005–2018
Source: Front Public Health. 2023 May 4;11:1174632. doi: 10.3389/fpubh.2023.1174632 (PMC10192628; doi:10.3389/fpubh.2023.1174632)
Supplement: Supplementary file 1 [file Table_1.DOCX]

**Table S1: Prevalence Ratios for contrasting diabetes prevalence in 2017-2018 vs 2005-2006 and percent reduction in β estimates in the models adjusted for alternative biological domain**^a^ **of risk factors (N=16 091)**

| **Models** | **Prevalence Ratio (95% CI)** | **Percent Reduction in β (95% CI), %**^b^ |
| --- | --- | --- |
| Base model | 1.40 (1.14-1.72) | [Reference] |
| **Individual adjustment for each of the other biological risk factors** |  |  |
| Diastolic blood pressure | 1.41 (1.15-1.73) | -1.8 (-7.7 to 2.6) |
| Uric acid | 1.40 (1.14-1.71) | 0.5 (-7.2 to 5.7) |
| Estimated glomerular filtration rate | 1.49 (1.23-1.81) | -19.0 (-43.1 to -6.5) |
| **Individual adjustment for the alternative biological domain** |  |  |
| Base + biological domain | 1.24 (1.05-1.46) | 36.8 (11.6 to 68.7) |
| **Sequential adjustment for the alternative biological domain** |  |  |
| Base + genetic + demographic + SDOH + lifestyle + obesity + biological domain | 1.00 (0.86-1.16) | 99.8 (64.3 to 175.3) |
| Further including psychosocial domain (full model) | 1.00 (0.86-1.16) | 100.8 (66.1 to 174.4) |

^a^ The alternative biological domain included 8 risk factors (systolic blood pressure, taking angiotensin-converting enzyme inhibitors, taking angiotensin II receptor blockers, taking β blockers, taking thiazides, total cholesterol, high-density lipoprotein cholesterol, and taking statins) from the primary biological domain and additional three risk factors (diastolic blood pressure, uric acid, and estimated glomerular filtration rate).

^b^ Percent reduction in the β coefficient, an estimate to quantify the percent contribution of individual and collective risk factors to the increasing prevalence of diabetes comparing 2017-2018 to 2005-2006, was obtained through contrasting the two models under comparison: (β_ref_-β_adj_)/β_ref_*100%. β_ref_ was based on the base model which is a crude Poisson model not adjusted for any domains of risk factors. β_adj_ was based on the model including one or more risk factors compared with the base model. The 95% confidence intervals (95% CIs) were estimated by performing bootstrap resampling (n=200). To conservatively account for possible non-linear associations between risk factors and diabetes, a quadratic term was added for all the risk factors in continuous form.

**Table S2. Prevalence ratios for contrasting diabetes prevalence in 2017-2018 vs 2005-2006 and percent reduction in β estimates according to individual risk factors (N=12 586)**

| **Risk Factors** | **Prevalence Ratio (95% CI)** | **Percent Reduction in β (95% CI), %**^a^ |
| --- | --- | --- |
| Base model | 1.46 (1.12-1.90) | [Reference] |
| **Individual adjustment for each risk factor** |  |  |
| Family history of diabetes | 1.38 (1.08-1.76) | 14.5 (2.5 to 35.8) |
| Age | 1.36 (1.07-1.71) | 19.4 (4.7 to 44.7) |
| Sex | 1.46 (1.12-1.90) | 0.2 (-2.4 to 2.9) |
| Race and ethnicity | 1.43 (1.10-1.86) | 5.4 (1.6 to 12.0) |
| Marital status | 1.48 (1.14-1.92) | -3.9 (-14.9 to 3.4) |
| Education level | 1.52 (1.17-1.97) | -10.5 (-28.0 to -2.8) |
| Employment status | 1.41 (1.12-1.78) | 8.7 (-1.9 to 24.8) |
| Ratio of family income to poverty | 1.44 (1.10-1.89) | 2.9 (-2.2 to 9.2) |
| Country of birth | 1.45 (1.11-1.90) | 1.1 (0.1 to 3.2) |
| Total number of people in the household | 1.46 (1.12-1.90) | 0.1 (-5.8 to 6.2) |
| Food security | 1.41 (1.08-1.85) | 8.7 (3.2 to 22.6) |
| Health insurance type | 1.34 (1.03-1.75) | 21.5 (12.2 to 42.6) |
| Routine place to go for healthcare | 1.50 (1.16-1.95) | -8.2 (-20.2 to -3.3) |
| Healthy Eating Index 2015 score | 1.47 (1.12-1.91) | -1.4 (-5.2 to 0.9) |
| Leisure-time physical activity | 1.37 (1.06-1.79) | 15.9 (6.3 to 34.1) |
| Cigarette smoking | 1.48 (1.14-1.92) | -3.5 (-13.8 to 4.9) |
| Alcohol consumption | 1.52 (1.17-1.98) | -10.9 (-27.1 to 0.3) |
| Sleep hours at night | 1.42 (1.09-1.85) | 6.9 (-2.5 to 18.7) |
| Body mass index | 1.36 (1.05-1.75) | 19.3 (5.1 to 38.8) |
| Waist circumference | 1.28 (1.01-1.64) | 33.6 (16.6 to 64.6) |
| Systolic blood pressure | 1.43 (1.13-1.81) | 5.0 (-9.9 to 17.9) |
| Taking angiotensin-converting enzyme inhibitors | 1.46 (1.15-1.86) | -0.5 (-21.3 to 15.9) |
| Taking angiotensin II receptor blockers | 1.42 (1.11-1.82) | 7.0 (-2.8 to 19.2) |
| Taking β blockers | 1.34 (1.05-1.72) | 21.9 (7.9 to 47.1) |
| Taking thiazides | 1.48 (1.16-1.89) | -3.5 (-17.8 to 5.2) |
| Total cholesterol | 1.39 (1.06-1.81) | 13.4 (5.3 to 27.0) |
| High-density lipoprotein cholesterol | 1.39 (1.07-1.81) | 12.4 (1.8 to 27.9) |
| Taking statins | 1.28 (0.99-1.65) | 35.3 (17.9 to 68.2) |
| Hours worked last week | 1.42 (1.12-1.81) | 6.8 (-3.5 to 20.1) |
| Depression score | 1.42 (1.09-1.85) | 7.6 (3.8 to 17.6) |
| Have trouble sleeping | 1.39 (1.06-1.81) | 13.4 (6.0 to 26.8) |

^a^ Percent reduction in the β coefficient, an estimate to quantify the percent contribution of individual risk factors to the increasing prevalence of diabetes comparing 2017-2018 to 2005-2006, was obtained through contrasting the two models under comparison: (β_ref_-β_adj_)/β_ref_*100%. β_ref_ was based on the base model which is a crude Poisson model not adjusted for any domains of risk factors. β_adj_ was based on the model including individual risk factors compared with the base model. The 95% confidence intervals (95% CIs) were estimated by performing bootstrap resampling (n=200). To conservatively account for possible non-linear associations between risk factors and diabetes, a quadratic term was added for all the risk factors in continuous form.

**Table S3. Prevalence ratios for contrasting diabetes prevalence in 2017-2018 vs 2005-2006 and percent reduction in β estimates according to individual domains (N=12 586)**

| **Models** | **Prevalence Ratio (95% CI)** | **Percent Reduction in β (95% CI), %**^a^ |
| --- | --- | --- |
| Base model^b^ | 1.46 (1.12-1.90) | [Reference] |
| **Individual adjustment for each domain** |  |  |
| Base + genetic domain | 1.38 (1.08-1.76) | 14.5 (2.5 to 35.8) |
| Base + demographic domain | 1.28 (1.02-1.61) | 34.1 (17.1 to 72.3) |
| Base + social determinants of health domain | 1.45 (1.15-1.84) | 1.3 (-18.1 to 17.9) |
| Base + lifestyle domain | 1.44 (1.11-1.86) | 4.2 (-11.5 to 22.5) |
| Base + obesity domain | 1.28 (1.01-1.62) | 35.4 (18.6 to 69.7) |
| Base + biological domain | 1.25 (1.01-1.53) | 41.6 (19.3 to 80.8) |
| Base + psychosocial domain | 1.36 (1.06-1.74) | 18.8 (7.5 to 40.8) |
| **Sequential adjustment for each domain** |  |  |
| Base + genetic domain | 1.38 (1.08-1.76) | 14.5 (2.5 to 35.8) |
| Further including demographic domain | 1.21 (0.98-1.50) | 48.9 (25.2 to 93.3) |
| Further including social determinants of health domain | 1.22 (0.98-1.51) | 48.0 (21.5 to 92.0) |
| Further including lifestyle domain | 1.19 (0.96-1.48) | 53.9 (29.4 to 113.2) |
| Further including obesity domain | 1.15 (0.94-1.40) | 64.0 (33.1 to 120.4) |
| Further including biological domain | 1.06 (0.87-1.29) | 84.4 (50.7 to 153.3) |
| Further including psychosocial domain | 1.02 (0.87-1.28) | 85.5 (49.9 to 156.1) |
| **Adjustment for all domains but excluding 1 domain** |  |  |
| Excluding genetic domain | 1.08 (0.89-1.32) | 79.2 (48.4 to 146.0) |
| Excluding demographic domain | 1.13 (0.93-1.38) | 67.9 (37.1 to 118.1) |
| Excluding social determinants of health domain | 1.05 (0.87-1.28) | 86.2 (51.5 to 157.5) |
| Excluding lifestyle domain | 1.06 (0.87-1.28) | 85.2 (52.4 to 153.2) |
| Excluding obesity domain | 1.08 (0.88-1.33) | 79.7 (47.1 to 146.8) |
| Excluding biological domain | 1.13 (0.92-1.38) | 67.5 (36.4 to 127.3) |
| Excluding psychosocial domain | 1.06 (0.87-1.29) | 84.4 (50.7 to 153.3) |
| **Adjustment for non-modifiable and modifiable domains** |  |  |
| Base + non-modifiable domains | 1.21 (0.98-1.50) | 48.9 (25.2 to 93.3) |
| Base + modifiable domains | 1.16 (0.94-1.42) | 61.1 (33.7 to 112.2) |

^a^ Percent reduction in the β coefficient, an estimate to quantify the percent contribution of individual and collective domains of risk factors to the increasing prevalence of diabetes comparing 2017-2018 to 2005-2006, was obtained through contrasting the two models under comparison: (β_ref_-β_adj_)/β_ref_*100%. β_ref_ was based on the base model. β_adj_ was based on the model including one or more risk factor domains compared with the base model. The 95% confidence intervals (95% CIs) were estimated by performing bootstrap resampling (n=200). To conservatively account for possible non-linear associations between risk factors and diabetes, a quadratic term was added for all the risk factors in continuous form.

^b^ Base model is the crude Poisson model not adjusted for any domains of risk factors.
